# Assessment of Properties of Structural Lightweight Concrete with Sintered Fly Ash Aggregate in Terms of Its Suitability for Use in Prestressed Members

**DOI:** 10.3390/ma16155429

**Published:** 2023-08-02

**Authors:** Małgorzata Rodacka, Lucyna Domagała, Rafał Szydłowski

**Affiliations:** Faculty of Civil Engineering, Cracow University of Technology, 31-155 Cracow, Poland; lucyna.domagala@pk.edu.pl (L.D.); rafal.szydlowski@pk.edu.pl (R.S.)

**Keywords:** lightweight concrete, lightweight aggregate, sintered fly ash, compressive strength, tensile strength, modulus of elasticity, shrinkage, creep, prestressed beams

## Abstract

The main aim of the paper was to assess whether the lightweight concrete with a new type of sintered fly ash aggregate can be used as a structural material for post-tensioned elements subject to high effort. This purpose was achieved by comparison of the properties of lightweight aggregate concrete with Certyd aggregate (LWAC) and normal-weight concrete with dolomite aggregate (NWAC) of similar strength in terms of their suitability for use in prestressed members. Special emphasis was placed on long-term, relatively rarely performed tests of rheological properties such as shrinkage and creep. The research was conducted on standard specimens as well as on plain and post-tensioned beams of bigger scale, which could reflect better the behavior of the materials in a destined type of structural members. The carried out tests showed that, despite the expected lower density and modulus of elasticity, LWAC revealed comparable tensile strength and lower shrinkage and creep in the whole time of observations (ca 1.5 years) in comparison to NWAC. Moreover, the total loss of prestressing force for beams made of LWAC was slightly lower than for NWAC. Estimations of tensile strength and modulus of elasticity values according to the standard Eurocode EN-1992-1-1 for both concrete types turned out to be satisfactory. However, the rheological properties of the tested lightweight concrete seemed to be considerably overestimated.

## 1. Introduction

Lightweight aggregate concrete (LWAC) as a structural material was already used in ancient times. The technology of this type of cement composite was developed by the Romans, who applied concrete with crushed bricks or volcanic rock aggregates such as pumice to construct both buildings and engineering facilities. One of the most famous and admirable buildings from that time that has survived to this day is the Pantheon in Rome. In modern times, the first structural application of lightweight concrete made of manufactured aggregate was in 1928, when LWAC was used to add eight additional floors to a skyscraper of Bell Telephone Company in Kansas City [1,2]. Currently, lightweight aggregates (LWAs) with better mechanical properties are available on the market, which allow for more favorable concrete parameters.

According to standard EN-1992-1-1 [3], structural lightweight concrete is defined as concrete with a minimum strength class of LC12/13 and its oven-dry density not less than 800 kg/m^3^ and not more than 2000 kg/m^3^. This concrete should be characterized by a closed structure and be made with the use of only or partly lightweight aggregate of mineral origin. In comparison to normal-weight concrete (NWAC), such lightweight concrete is usually characterized by greater structural homogeneity resulting from a more similar modulus of elasticity of the aggregate and the cement matrix, their better mutual adhesion, and more uniform shape and size of the manufactured LWA. Due to the different structures, lightweight aggregate concretes usually behave differently under load and show a different mechanism of failure compared to NWACs. In the case of LWAC, there are no three characteristic stages of crack development as in normal-weight concrete (I—formation of stable cracks, II—stable crack propagation, III—unstable crack propagation). For typical NWAC with rock aggregate subjected to compressive load, the stress limit between stage I and stage II is about 35–45% of concrete strength, while stage II changes to stage III at stress of about 70–90% of the compressive strength. Meanwhile, in concretes with lightweight aggregate, the first cracks appear even at the stress corresponding to 85–90% of the strength [4,5]. The tensile strength of lightweight concretes is more diverse, and its value depends more strongly on both the aggregate type and the test conditions. It is assumed that LWAC reveals lower tensile strength than NWAC of the same strength class [1,3,6]. This applies to all tested types of tensile strength: direct, splitting, and bending. Nevertheless, many studies on the tensile strength tests of structural lightweight concretes indicate a similar relationship between the tensile and compressive strengths as for normal-weight concrete, e.g., [5,7,8,9,10]. As Young’s modulus of concrete, which is a two-component composite, depends considerably on the modulus of elasticity of aggregate, LWAC is characterized by a much lower modulus of elasticity, which was confirmed in numerous studies [6,11,12,13,14,15]. In general, compared to normal-weight concretes of the same strength class, lightweight concretes can have the modulus of elasticity lower by 15–60% [5]. The reduction in LWAC modulus is dependent mainly on its density and the lightweight aggregate type. In designing structures, rheological properties of concrete, such as shrinkage and creep, are also important characteristics. Due to the lower modulus of elasticity of the porous aggregate, lightweight concretes are characterized by increased shrinkage in comparison to NWAC, even by up to 50%, which was confirmed by much research on this phenomenon [7,16,17,18]. On the other hand, since lightweight concretes have the ability to internal curing owing to water accommodated in LWA, the development of their shrinkage may be slower in time and less dependent on conditions of external curing such as temperature and humidity conditions. It was proved in numerous studies [19,20,21,22,23] that the shrinkage of high-strength concretes with lightweight aggregate was characterized by a slower increase even up to 1 year of curing, but later, it began to develop faster and finally reached the value by about 20% than the shrinkage of normal-weight concrete of the same strength. Generally, the experimental studies proved that in the case of lightweight concrete, like for normal-weight concrete, the following rule is applied: the higher the compressive strength, the lower the creep. Various research showed that LWAC creep can be higher [18] or similar [4,24,25,26] as for NWAC of comparable strength. In some rare cases, LWAC of higher strength class creep may be even lower than for NWAC of the same class [4].

The concept of using lightweight concrete for prestressed structures may turn out to be a very good alternative to normal-weight concrete. Due to the application of LWAC instead of NWAC, the amount of concrete and prestressing steel can be reduced [27]. Mayer and Khan [28] showed that the use of high-strength lightweight concrete allowed the design of load-bearing structures with a larger span. It was proved in [2,6,22,23,24,25] that achievement of LWAC of higher strength class, necessary for prestressed structural members, is not a problem. Nevertheless, in the available literature, there are not so many examples of research carried out on prestressed structural members due to the large size of such elements as well as complicated, long-term, and more expensive performance. The research dedicated to the application of structural lightweight concrete for the prestressed members is even rarer [26,27,28,29].

The aim of tests reported by Bymaster et al. in [29] was to compare the loss of the prestressing force in beams made of two types of lightweight concrete and one normal-weight concrete. The compressive strengths after 28 days were 51.5 MPa, 49.2 MPa, and 67.3 MPa for concrete with sintered shale, sintered clay, and granite aggregate, respectively. Beams with a cross-section of 165 × 305 mm and a length of 5.5 m were subjected to long-term loading for 75 days after 150 days from concreting. The prestressing force loss for lightweight concrete elements measured during these tests was very similar to the prestressing force loss for the beam made of normal-weight concrete.

Tests of prestressing force losses in beams made of various types of concrete were also carried out by Chen et al. [30]. In this case, normal-weight concrete with a 28-day strength of 50 MPa and two lightweight concretes with expanded clay and shale aggregate with a strength of 40–45 MPa were used to execute 3 m long beams with a box section (2877 × 1680 × 4637 mm) and a rectangular section (2100 × 2800 mm). The prestressing force was monitored for 180 days. In the case of lightweight concrete elements, the loss (5.4–6.8%) turned out to be lower than for normal-weight concrete beams (8.2–9.1%).

In turn, Vázquez-et al. [31] reported tests of prestressed concrete beams with a rectangular cross-section of 200 × 400 mm and a span of 3250–4000 mm, made of one NWAC with granite and two LWACs with DryArlita F7 lightweight aggregate. The concretes, after 28 days, reached a strength of 80, 70, and 75 MPa for normal-weight concrete and two lightweight concretes, respectively. The beams were subjected to a four-point bending. A greater load capacity of about 5–10% was observed for NWAC beams. However, the maximum deflection of LWAC beams was about 25% lower than that of beams made of normal-weight concrete. Moreover, the authors of the study observed that lightweight concrete beams failed more rapidly than normal-weight concrete beams.

Wu et al. [32] analyzed deflections of beams reinforced with steel fibers and prestressed with cables without adhesion made of carbon fiber reinforced polymer (CFRP). Beams with a cross-section of 400 × 200 mm were made of lightweight concrete with expanded slate aggregate and normal-weight concrete with strengths of approximately 45 MPa and 50 MPa, respectively. Eight beams with different spans of 3.6–4.8 m were made, two of LWAC and six of NWAC. Half of them were prestressed. All beams were destroyed during the four-point bending test. The prestressed beams achieved a 101% higher cracking moment. The lightweight concrete beams failed with less deflection and less breaking force. The increase in the force in the cables was lower, about 20%, for lightweight concrete beams than for normal-weight concrete members.

The main aim of the research was to assess whether lightweight concrete with a new type of sintered fly ash aggregate can be used as a structural material for post-tensioned elements subject to high effort. This purpose was achieved by comparison of the properties of LWAC and NWAC of similar strength in terms of their suitability for use in prestressed members. Due to the destiny of these concretes, special emphasis was placed on long-term tests of rheological properties such as shrinkage and creep specified on bigger elements and in much longer time than in the case of all studies referred above. As these characteristics have a direct impact on the value of rheological losses and deflections of prestressed elements, a longer time of observation could make the comparison more reliable. An additional purpose of this paper was also to verify the estimations of the structural properties values for lightweight and normal-weight concretes in accordance with the standard Eurocode EN-1992-1-1 [3]. Two types of concrete were made: NWAC with dolomite aggregate and LWAC on Certyd aggregate. Compressive strength, modulus of elasticity, tensile splitting strength, and long-term shrinkage and creep tests were carried out. The rheological tests were conducted on post-tensioned beams to observe the phenomena on test specimens of a bigger scale, which can better reflect the behavior of the materials in a destined type of structural members.

## 2. Materials and Methods

### 2.1. Materials

Tests were carried out on two types of concretes of comparable compressive strength levels suitable for prestressed members: lightweight aggregate concrete and normal-weight concrete as a reference composite.

**Figure 1:**
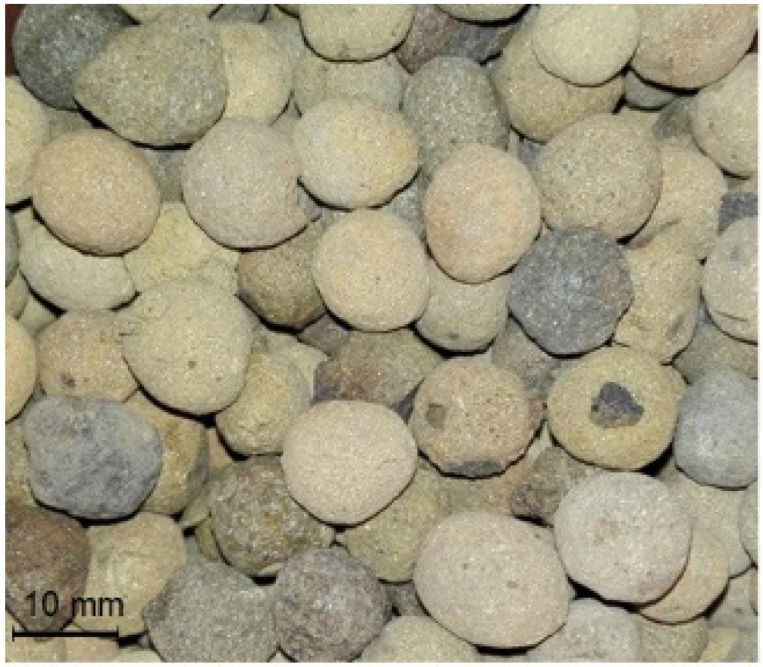
Certyd 4/16 mm—lightweight aggregate used for LWAC.

Lightweight concrete was made with Certyd aggregate (Figure 1). It is a new type of sintered fly ash LWA manufactured in Poland since 2015. Unlike expanded clays or shales, available on the market in various density types intended for a wide spectrum of concrete applications, from insulating to structural ones, Certyd is dedicated especially for structural purposes. Its particles are characterized by a closed structure of the external shell and a regular, spherical shape. The properties of the aggregate, shown in Table 1, especially crushing resistance and water absorption, indicate that in comparison to other sintered fly ash aggregates [1,2,6,14,18], this LWA may create new opportunities for its application in more advanced fields, e.g., for prestressed structures. Due to its high water absorption the aggregate was initially prewetted to its moisture content corresponding to its water absorption after 24 h (WA_24h_) before application for concrete. Such a procedure protected fresh concrete from workability loss and too high cement content.

Normal-weight concrete was made of dolomite coarse crushed aggregate. Its rough texture and uniform structure enabled the formation of a very good bond between the aggregate and the cement matrix. Owing to better concrete homogeneity, this type of dolomite aggregate has typically been used for structural composites, especially for prestressed structures such as viaducts and bridges. The properties of the used dolomite are compared with lightweight Certyd in Table 1.

The rest of the constituent materials applied for the production of both concretes were natural quartzite sand, Portland cement CEM I 42.5R, potable water, and superplasticizer. The compositions of lightweight and normal-weight concretes were designed in such a way that both composites had the same consistency in fresh conditions (V4/V5) and comparable strength in the hardened stage (45–55 MPa). Additionally, lightweight concrete density was assumed to be classified as D1.8. As a result, the composition of LWAC was characterized by a higher content of cement and superplasticizer and a lower nominal water–cement coefficient in comparison to NWAC. Compositions of both concretes are presented in Table 2. The normal-weight concrete was prepared about 3 months later than the lightweight composite when the development of LWAC strength was known, and it was possible to design NWAC of comparable strength.

### 2.2. Methods

The consistency of the fresh concretes was tested according to EN 12350-5 [33], as the Vebe method is the most reliable test for LWAC. The density of the fresh concretes was examined according to EN 12350-6 [34]. Then, specimens for all planned tests were molded. The types of hardened concrete tests to be carried out, suitable standard procedures, and the type and number of test specimens are listed in Table 3.

#### 2.2.1. Tests of Hardened Concretes Specified on Standard Specimens

Tests of density, compressive strength, modulus of elasticity, and tensile splitting strength were carried out according to European Standards procedures [36,37,38,39]. All standard test specimens were made, maintained, and stored in accordance with EN 12390-2 [39] and EN 12390-1 [40]. They were demolded after 24 h from casting and stored in a climatic chamber, where a constant temperature of 20 °C and 100% humidity was maintained. All the standard measurements were carried out at concrete ages 14, 28, and 90 days. For each type of concrete, particular mechanical properties at a given age were tested on 6 specimens. Density tests were carried out in three conditions: saturated, natural, and oven-dried on three specimens at each concrete age. The oven dry density was measured after the determination of the density in saturated condition on the same set of specimens. The natural density was measured on specimens stored together with beams intended for rheological tests. The total number of prepared standard specimens for both LWAC and NWAC was 108.

#### 2.2.2. Tests of Hardened Concretes Specified on Big-Scale Beams

**Figure 2:**
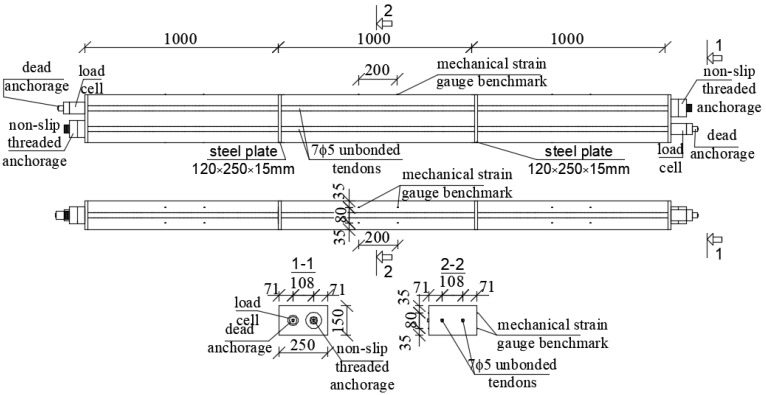
Scheme of beams for shrinkage and creep testing with the arrangement of prestressing cables and sensors for measuring deformations.

**Figure 3:**
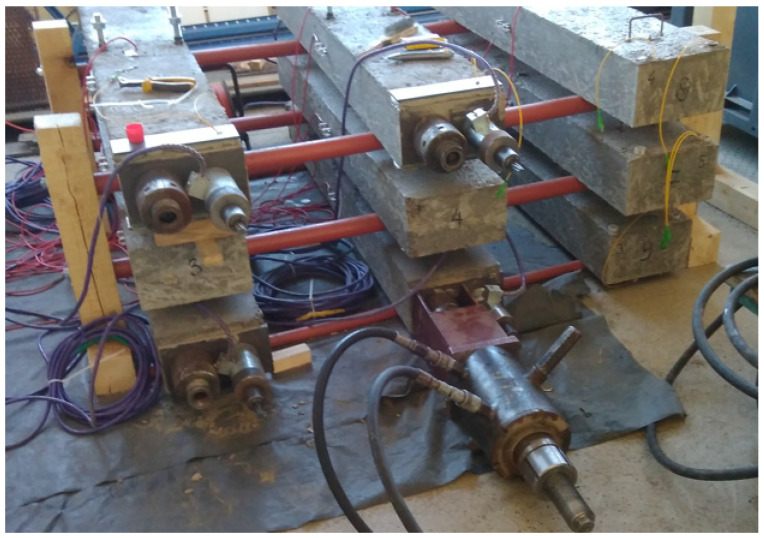
View of the beams for shrinkage and creep testing.

**Figure 4:**
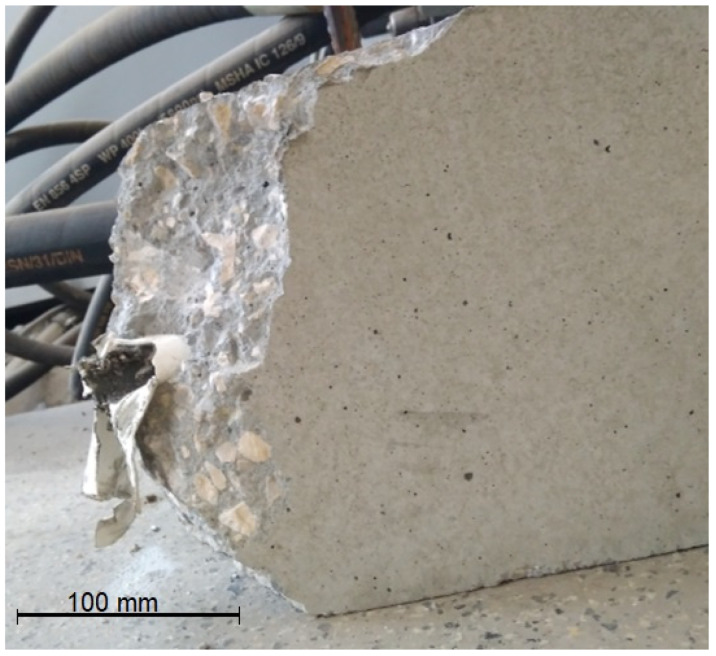
Damaged normal-weight concrete beam for creep and shrinkage tests.

In the case of testing, shrinkage and creep much bigger than typical standard specimens, i.e., beams 200 × 200 × 1000 mm, were used. For each concrete type, six beams were cast: three massive beams and three with inner ducts formed for post-tensioning. In the middle of the length of each beam, two 200 mm long measuring bases were installed on opposite sides to measure deformations. The scheme of beams for shrinkage and creep testing with the arrangement of prestressing cables and sensors for measuring deformation is given in Figure 2. Shrinkage measurements were started on the next day after beams concreting. At the age of 14 days, three beams of each concrete type were prestressed with unbonded tendons to introduce compressive stress. The strands of 7ϕ5 mm were inserted into the ducts of 3 beams connected together with the layer of 15 mm thick mortar (Figure 2 and Figure 3). The value of the stress introduced into the cross-section of the beams was about 10.0 MPa. We used 15 mm thick steel retaining plates and glued them onto the faces of the connected beams. On the tension side, a non-slip active (bolt) anchorage was used. Under the passive anchorage, Gecon 4700 string force transducers were installed. During the prestressing of NWAC beams, one of them failed at the cable anchorage. The destruction occurred by crushing the concrete. Analyzing the possible reasons for the failure, incorrect compaction of the concrete was excluded because the failure cross-section showed neither segregation nor structural discontinuity. Lower local compressive strength can be indicated as the most probable cause of the element failure. Figure 4 presents a photo of the damaged beam. Finally, tests of rheological properties of normal-weight concrete were carried out on five beams.

Strain readings were taken with the mechanical sensor Demec over a period of 559 days for lightweight concrete and 489 days for normal concrete. The difference in measuring time for LWAC and NWAC beams resulted from different times of execution of these beams (see Section 2.1). For each type of concrete, the shrinkage deformations were obtained by calculating the average value for each of the two measurements from all three non-prestressed beams. However, creep strain was calculated by subtracting the average strains measured on prestressed beams from the average strains measured on plain beams.

## 3. Results

### 3.1. Results and Discussion

#### Consistency and Density of Fresh Concrete

For both LWAC and NWAC, the Vebe time was 5–6 s. According to National Annex PN-B-06265 [41] to EN 206 [42], this result is the basis for classifying the consistency of both mixtures as V4/V5. The constant consistency was maintained throughout the concreting period of the specimens and beams. Both concrete mixes showed no tendency to segregate during compaction with a poker vibrator.

The density of fresh LWAC was 1990 kg/m^3^, while in the case of NWAC, it was 2330 kg/m^3^.

### 3.2. Density of Hardened Concrete

Mean densities of hardened concretes in saturated, natural, and oven-dry conditions at 28 days are presented in Table 4.

The obtained density of lightweight concrete in a dry condition classifies it according to EN 206 [42] to density class D1.8. It corresponds to the assumption made in designing concrete composition. The density of normal-weight concrete was 25% higher than for LWAC. Water absorption, calculated on the basis of density measurements in a dry and saturated condition, was 13.8% and 6.9%, respectively, for lightweight and normal-weight concrete. However, the moisture content of specimens when stored at a temperature of 20 °C and relative humidity of about 50% was 8.6% and 4.6%. The value of water absorption for LWAC, although twice as high as for NWAC, can be assessed as moderate. According to [4], water absorption of typical structural lightweight concretes ranges from 5% to 25%. The standard guidelines PN-91/B-06263 [10] recommend that structural lightweight concrete exposed directly to weather conditions should have water absorption lower than 20% and, in the case of protection, lower than 25%.

### 3.3. Compressive Strength

**Figure 5:**
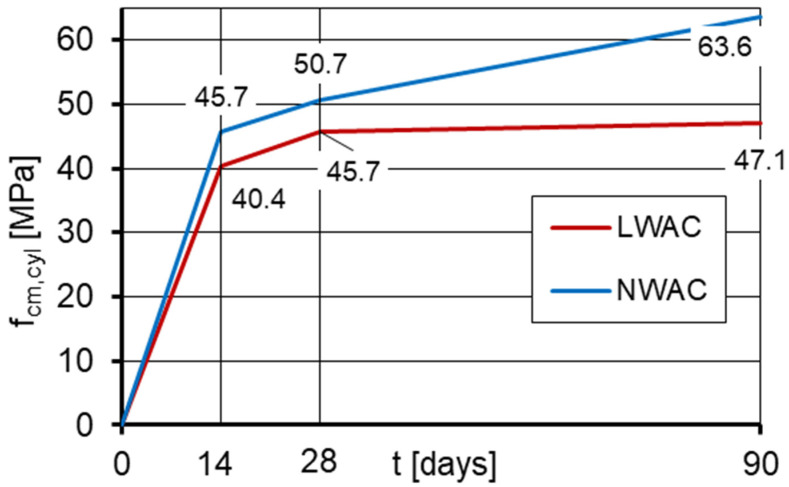
The development of compressive strength in time determined on LWAC and NWAC cylindrical specimens.

The mean values of compressive strength determined at the age of 14, 28, and 90 days on standard cylindrical specimens are shown in Figure 5. The average compressive strength specified on standard cube specimens at 28 days was 48.5 and 50.7 MPa, respectively, for lightweight concrete and normal-weight one. The coefficient of variation for compressive strength, defined as a ratio of a standard deviation and a mean value, ranged from 0.8% to 3.5% for LWAC and from 0.6% to 4.5% for NWAC. These comparable and relatively low values of the coefficient prove very good structural homogeneity of both concretes.

Comparing the test results after 28 days, it should be stated that, regardless of the type of specimens used for the strength determination, similar average compressive strengths were obtained for both concretes, and their values corresponded to the assumptions. The average compressive strength of normal-weight concretes was only 4% and 11% higher than that of lightweight concretes, respectively, when determined on cubic and cylindrical specimens.

Nevertheless, the strength development of both concretes over time turned out to be different, especially in ages later than 28 days. Between the age of 28 and 90 days, NWAC showed a significant 25% increase in strength, while during this time, LWAC strength increased only by 3%. A negligible increase in the compressive strength of lightweight concrete after 28 days is due to the limiting effect of lightweight aggregate. The low strength of LWA, which is the weakest component of the concrete, prevented the use of the potential of a stronger cement matrix in the further development of the composite’s strength. Such observations regarding the strength development of lightweight aggregate concretes are consistent with the research reported in [4,14].

Small differences between the compressive strength determined on cubic (f_cm,cube_) and cylindrical specimens (f_cm,cyl_) indicate very good structural homogeneity of both concretes. In the case of typical structural normal-weight concretes and lightweight concretes, their compressive strength at 28 days determined on cylinders is usually lower than the strength tested on cubes by about 20% and 10%, respectively [4,5]. Meanwhile, the ratio of f_cm,cyl_ to f_cm,cube_ for the tested LWAC was 6%, while the tested NWAC showed no scale effect et al., which was probably caused by the application of a very homogeneous dolomite aggregate with a rough texture. Such results confirm the reports [4,6] that in the case of concretes characterized by significant structural homogeneity, the assessment of concrete strength specified on cylindrical specimens instead of cubes may lead to a higher strength class of composite. Finally, in the case of tested concretes, despite almost the same mean cube strength, according to EN 206 [42], LWAC may be classified as LC40/43 while NWAC as C45/55.

**Figure 6:**
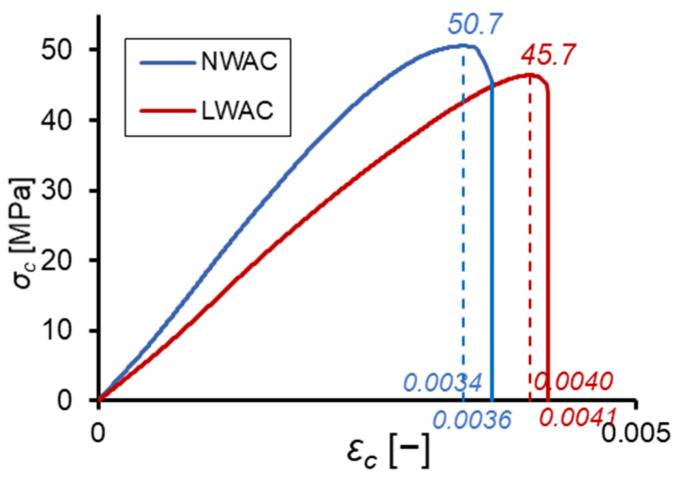
Stress—strain relationship in the uniaxial compression test at 28 days for lightweight and normal-weight concretes.

The average stress–strain relationships for lightweight and normal-weight concretes in the uniaxial compression test at 28 days are shown in Figure 6. For LWAC, the diagram has a rectilinear form up to about 80% effort. This result confirms the reports [1,4,5,43], which showed that due to the lower stress concentration in the contact zone between the lightweight aggregate and the cement matrix, LWAC may work under load in the non-cracked condition to much higher stress levels than NWAC. In the case of tested normal-weight concrete, the stress–strain relationship was rectilinear up to about 65% of its strength, which is much higher than the limit stress typical for structural NWAC [4,5]. However, compared to the tested normal-weight concrete, LWAC failed faster after reaching strength, indicating its more brittle nature, as shown by the smaller area under the descending part of the stress–strain diagram.

Besides the low values of the coefficient of variation and the rectilinearity of the stress–strain relationship to the relatively high level of stress, the additional proof of the very good structural homogeneity of both concretes is their way of failure in the compression test. All specimens of both LWAC and NWAC were damaged by coarse aggregate particles, not by the contact zone between the aggregate and cement paste. Such a way of failure proves a very good bond of the matrix not only to the porous lightweight aggregate but also to the natural dolomite crushed aggregate. In the case of normal-weight concrete, this is not a typical form of failure [4,5].

### 3.4. Tensile Splitting Strength

**Figure 7:**
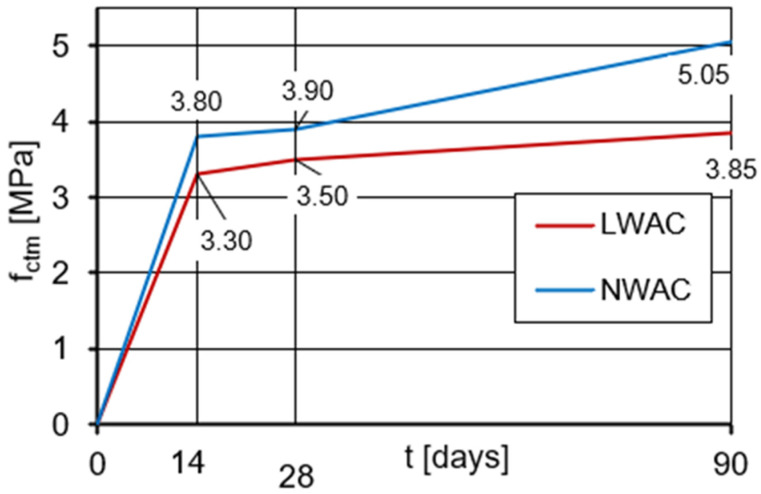
The development of tensile strength in time determined on LWAC and NWAC cylindrical specimens.

The mean values of tensile splitting strength (f_ctm_) determined at the age of 14, 28, and 90 days on standard cylindrical specimens are shown in Figure 7. The coefficient of variation for tensile strength ranged from 1.9% to 10.5% for LWAC and from 4.7% to 11.7% for NWAC. These values, although much higher than in the case of the compressive strength test, for the tensile strength test shall be assessed as relatively low due to the test specificity.

**Figure 8:**
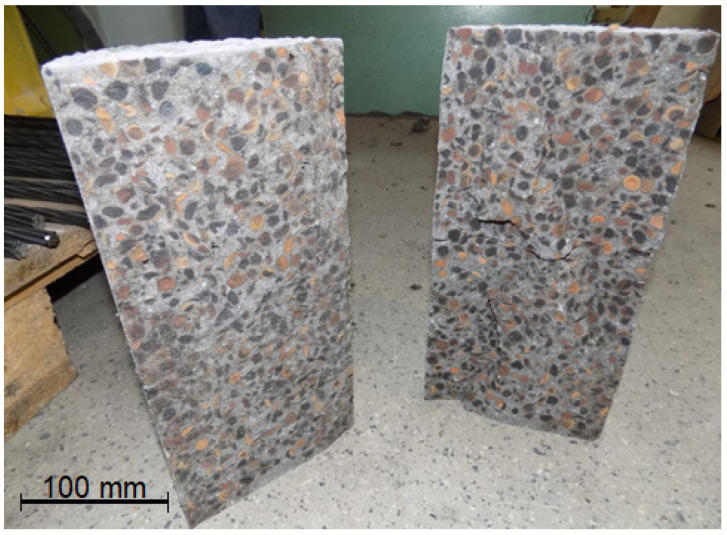
Lightweight concrete specimens split during tensile splitting test.

**Figure 9:**
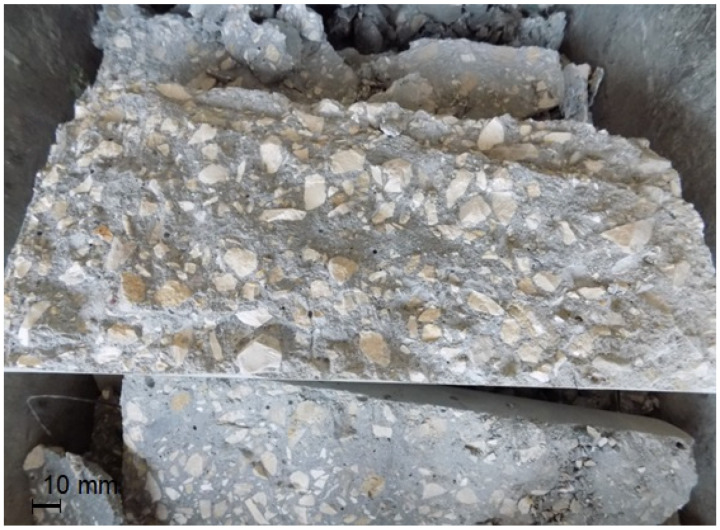
Normal-weight concrete specimens split during tensile splitting test.

The tensile strength of lightweight concrete after 28 days of curing was 11% lower than that of normal-weight concrete, similar to the 28-day compressive strength. The presented results confirm that the tensile splitting strength value of lightweight concrete is very close to the tensile strength value of normal-weight concrete of similar compressive strength [5,7,44]. In the case of both tested concretes, the ratio of f_ctm_/f_cm_ was 8%. The lower increase in tensile strength between 28 and 90 days of curing for lightweight concrete, as in the case of compressive strength, is due to the limiting effect of the weak porous aggregate. In Figure 8, there is a photo of the lightweight concrete specimens damaged during the splitting tensile test, which presents typical failure for LWAC—via the aggregate particles. In the case of normal-weight concrete (Figure 9), a similar effect was observed. The above observation once again confirms the very good adhesion of cement paste to the aggregate in the case of both tested concretes.

According to EN-1992-1-1 [3], the axial tensile strength value can be estimated as 0.9 of the splitting tensile strength value. For the concrete of class LC 40/43 and the dry density of 1740 kg/m^3^, the value of the average axial tensile strength is assumed as 3.05 MPa, which gives the tensile splitting strength estimated as 3.4 Mpa. Therefore, the calculated value is lower than the one measured after 28 days by only 3%. In the case of normal-weight concrete of class C45/55, the splitting tensile strength according to EN-1992-1-1 [3] can be estimated at 4.2 Mpa. Meanwhile, the measured value was 3.90 MPa, which is lower by 7% than the estimated value. Small differences between the values calculated in accordance with EN-1992-1-1 [3] and the values measured for both lightweight and normal concrete indicate a reliable standard estimation.

### 3.5. Modulus of Elasticity

**Figure 10:**
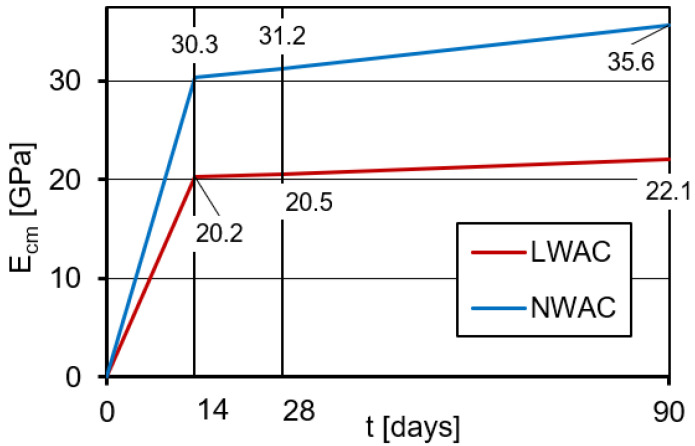
The development of modulus of elasticity in time determined on LWAC and NWAC cylindrical specimens.

The mean values of the secant modulus of elasticity (E_cm_) determined at the age of 14, 28, and 90 days on standard cylindrical specimens are shown in Figure 10. The coefficient of variation for modulus of elasticity ranged from 3.4% to 4.2% for LWAC and from 2.1% to 5.0% for NWAC.

The results of the carried out tests are consistent with numerous studies [4,11,12,13,14,15], in which it was indicated that the modulus of elasticity of lightweight concrete was significantly lower than for normal-weight concretes with a similar average compressive strength. In the tested case, LWAC modulus at 28 days was lower by as much as 34% in comparison to NWAC at this age.

According to EN-1992-1-1 [3], the estimated value of the modulus of elasticity for lightweight concrete of strength class LC40/43 and the density in the dry condition of 1740 kg/m^3^ is 21.9 GPa. This value is 6% higher than the modulus value determined in tests after 28 days. The modulus of elasticity of normal-weight concrete of class C45/55, made of dolomite aggregate, is estimated in accordance with EN-1992-1-1 [3] for 32.4 GPa. The calculated value of the modulus of elasticity was only 4% higher than that tested after 28 days. These small differences between the specified and the standard values indicate a reliable estimation of the modulus of elasticity according to EN-1992-1-1 [3] for both lightweight and normal-weight concrete.

### 3.6. Shrinkage

**Figure 11:**
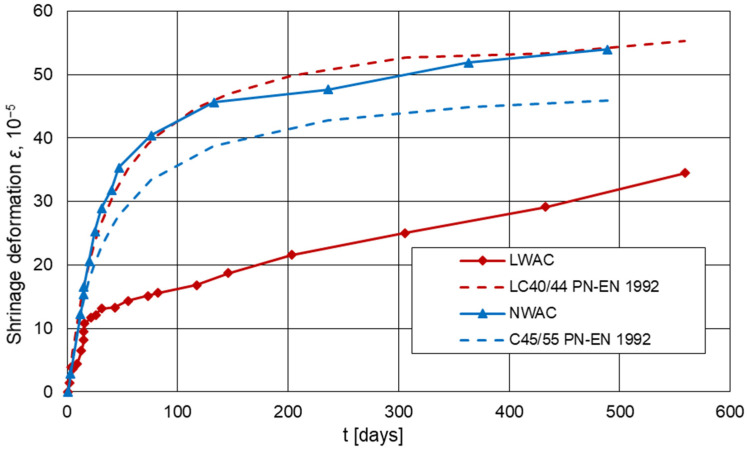
Development of average deformation from shrinkage, tested and estimated in accordance with EN 1992-1 [3], in time for lightweight and normal-weight concrete (approx. RH = 50%, approx. T = 20 °C).

Figure 11 presents diagrams of the development of shrinkage deformations for lightweight and normal-weight concrete, as well as the development of shrinkage calculated in accordance with EN-1992-1-1 [3] for concretes of classes C45/55 and LC40/44. The coefficient of variation for shrinkage ranged from 2.1% to 13.8% for LWAC and from 2.0% to 4.6% for NWAC. Much higher coefficient values for LWAC were observed at the initial period of concrete drying due to its considerably higher moisture.

The obtained charts indicate significant differences in the values and in the development of shrinkage deformations over time for lightweight and normal-weight concretes. The initial, more dynamic increase in shrinkage strains for lightweight concrete lasted about 15 days; in the case of normal-weight concrete, it lasted about 50 days. The slowdown of the dynamics of the increase in shrinkage deformations started from the value of approximately 9 × 10^−5^ and 45 × 10^−5^ for lightweight and normal-weight concretes, respectively. Significantly lower shrinkage of lightweight concrete was caused by the ability of porous aggregate to accommodate large amounts of water. As a result, there was probably a reduction in initial shrinkage by eliminating the self-drying of lightweight concrete caused by cement hydration and limiting water evaporation from the composite. In the next stage of shrinkage development, a linear increase in deformation is observed for both concretes. However, It is worth noting that the strain increment for lightweight concrete is greater and did not stabilize as opposed to normal-weight concrete. The lack of stabilization indicates the possibility of achieving greater shrinkage of lightweight concrete in the later period. After 559 days, the shrinkage value of lightweight concrete was 34.5 × 10^−5^, and for normal-weight concrete after 469 days was 54 × 10^−5^. The final shrinkage value of LWAC is as much as 36% lower than the shrinkage value of NWAC. Despite testing the shrinkage for about 1.5 years, the values for lightweight concrete were still significantly lower than for normal-weight concrete.

The course and shrinkage values for lightweight concrete differ significantly from the values calculated according to EN-1992-1-1 [3] for classes LC40/43. According to the standard, shrinkage of lightweight concrete should increase faster in the initial period and then stabilize. In the presented studies, the period of faster growth was shorter, and stabilization was not observed. The value of the calculated shrinkage after 559 days is 55.3 × 10^−5^, which is 138% of the measured values. However, it cannot be directly stated that the guidelines of the standard overestimate the results because, as mentioned above, the examined shrinkage still shows an upward trend. The lack of shrinkage stabilization may indicate that the shrinkage strain values may stabilize later and finally reach values close to those calculated in accordance with EN-1992-1-1 [3]. In order to establish the final shrinkage of lightweight concrete based on Certyd aggregate, the research should have been carried out until the stabilization of shrinkage deformations. On the other hand, a significant difference in the course of shrinkage development in the initial period between the tested and estimated values proves the lack of precise guidelines in EN-1992-1-1 [3] on the effect of initial wetting of the aggregate, due to which LWAC has the possibility of self-curing.

In the case of normal-weight concrete, the development of shrinkage deformations was more consistent with the predictions of the standard EN-1992-1-1 [3]. Comparing the results of the measured shrinkage deformations with the results calculated in accordance with EN-1992-1-1 [3], a very similar course of shrinkage development over time was obtained. However, the calculated shrinkage values are slightly lower than the measured ones. As a result, the final shrinkage value calculated according to EN-1992-1-1 [3] is 45.9 × 10^−5^ and is 15% lower than that tested for normal-weight concrete. The difference in values may be due to the fact that the NWAC strength after 28 days of curing was still significantly increasing. The average strength of normal-weight concrete after 28 days was 50.7 MPa, while after 90 days, it was 63.8 MPa.

The obtained test results for lightweight concrete confirm the results of studies presented in [19,20,21,22,45], which showed that the shrinkage of LWAC of higher strength was characterized by a slower increase in the initial stage of development than for normal-weight concrete. On the other hand, the further development of shrinkage deformations examined for lightweight concrete was not consistent with the studies [19,20,21,45]. In most tests, after about a year, the shrinkage of LWAC reached values similar to or higher than for NWAC. It should be noted that the cited tests were carried out on concretes made on other types of aggregates characterized by a different porosity structure and lower water absorption. The only exception was research by Costa et al. [20], where similar, low values of LWAC shrinkage were obtained. In the study referred [20], the shrinkage of lightweight concretes based on Leca aggregate was almost ten times lower than the standard predictions after 672 days. The authors explain this discrepancy by not taking into account the impact of lightweight concrete self-curing in the standard estimations. Additionally, attention should be paid that compared to the beams used in the above-cited studies [17,18,19,20,43], own research was carried out on much larger test elements, which can show more reliable behavior of concrete similar to this in construction.

However, it was confirmed in reported research [1] that concretes with sintered lightweight aggregate show relatively lower shrinkage than other lightweight concretes. Certyd is an aggregate sintered at high temperatures, which can have a beneficial effect on limiting concrete shrinkage. As the above test results and their discrepancies show, it is impossible to clearly define the development rule and shrinkage value of lightweight concretes. The analysis of the issue of lightweight concrete shrinkage requires taking into account not only the impact of the specificity and type of lightweight aggregate but also the composition of the concrete, with particular emphasis on the initial wetting of the aggregate.

### 3.7. Creep

**Figure 12:**
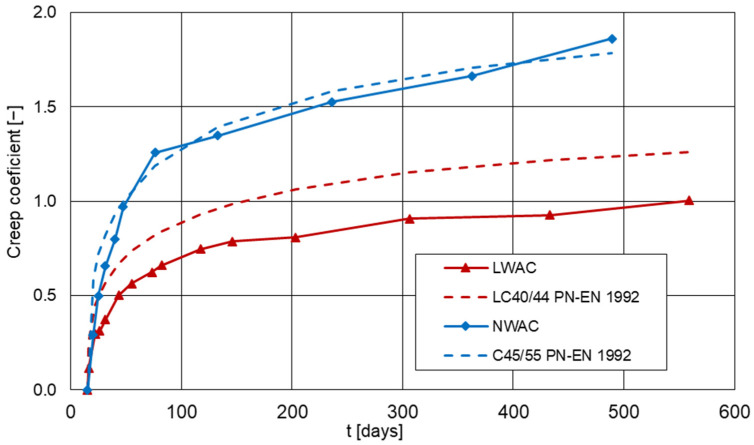
Development of average creep coefficient, tested and estimated in accordance with EN 1992-1 [3], in time for lightweight and normal-weight concrete (approx. RH = 50%, approx. T = 20 °C).

The charts of creep coefficient development in time, tested and calculated according to EN-1992-1-1 [3] for lightweight and normal-weight concretes are presented in Figure 12. The values of the measured creep coefficient were calculated according to the Formula (1).

(1) $$\varphi =\frac{\left(\varepsilon -\varepsilon_{0}\right)}{\varepsilon_{0}}$$

where:

φ—creep coefficient

ε—total strain

$\varepsilon_{0}$—elastic strain directly after unloading.

The coefficient of variation for creep ranged from 0.5% to 3.0% for LWAC and from 0.2% to 1.9% for NWAC.

Analyzing the course of the creep coefficient charts, it can be seen that in the case of both concretes, the most dynamic increase in the coefficient is observed in the period up to 100 days. At this age, the LWAC has reached a creep coefficient of approximately 0.7, while the NWAC reached 1.2. Differences in these values indicate a much greater increase in the creep of normal-weight concrete in the first several dozen days after loading. Then, for both concretes, a significant slowdown in the increase in the creep coefficient is observed. In the case of lightweight concrete, after about 200 days, a clear stabilization of the values can be seen, while for normal-weight concrete, the coefficient value increased until the end of the research. It can be expected that further NWAC testing would reveal a further increase in creep. The final creep coefficients registered in the tests were 0.87 and 1.86 for lightweight and normal-weight concrete, respectively. Therefore, the final value of creep deformations for LWAC was as much as 53% lower than for NWAC. This difference is probably due to the fact that in the tested lightweight concrete, the cement matrix had a higher strength compared to normal-weight concrete in order to compensate for the lower strength of the porous aggregate itself and ensure comparable strength of both composites. In addition, Certyd is the fly ash porous aggregate sintered at high temperatures, and the resulting hardened shell on the aggregate particles could advantageously reduce the creep. When analyzing the influence of the type of concrete on the value of creep, the fact that LWAC showed a higher moisture content than normal-weight concrete should not also be neglected.

It should also be noted that the results calculated according to EN-1992-1-1 [3] for lightweight concrete are significantly higher than those obtained from tests. Differences are observed primarily in the initial period of creep development, where, according to EN-1992-1-1 [3], it is faster and with higher values. The final creep coefficient calculated according to EN-1992-1-1 [3] is 1.26 and is 31% higher than the tested one. The standard EN-1992-1-1 [3] reduces the creep value for lightweight concretes of class greater than LC20/22 due to the higher strength of the cement matrix but does not take into account the type of aggregate used and its initial moistening. For this reason, the calculated values may be overestimated. On the other hand, for normal-weight concrete, the calculated values are very similar to those obtained from the tests, and a practical agreement between the calculated and measured values was achieved. The final value of the creep coefficient calculated in accordance with EN-1992-1-1 [3] for normal concrete is 1.78 and is only 4% lower than the tested value.

The obtained test results are consistent with those presented in [17,19,21,46,47], where the creep of lightweight concrete was lower than that of normal-weight concrete. It should be emphasized that in the discussed publications, the LWAC creep was lower by about 5–30% than the creep of NWAC, while in this research, the creep of lightweight concrete was as much as 50% lower than for normal weight. Only in one study [17] were similar results achieved, where after 10 days, the creep was 50% of the final value, and after 250 days, 90%.

**Figure 13:**
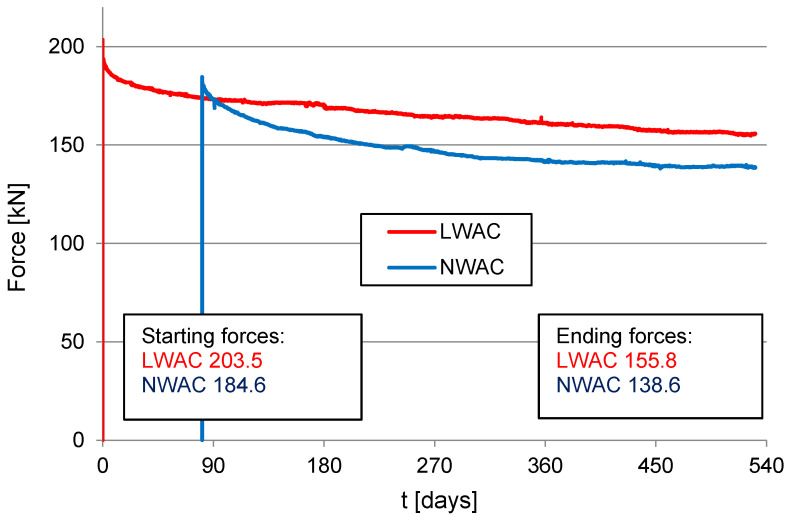
The change of the prestressing force in the cables of prestressed beams in time.

Figure 13 shows the relationship of prestressing force in time for lightweight and normal-weight concrete beams. The total loss of prestressing force was 23% and 25% for LWAC and NWAC, respectively. A slightly lower loss of prestressing force for LWAC was also reported in [29,30]. Such results indicate that in the case of the application of LWAC, rheological phenomena may affect the behavior of prestressed elements in time less than for structural members made of NWAC. Nevertheless, to prove this thesis, it is necessary to test full-scale prestressed members.

## 4. Conclusions

The carried out research program and the conducted analyses give the basis for formulating the following conclusions:

- The average density of the tested lightweight concrete was 20% lower than that of normal-weight concrete, while its average compressive strength at 28 days was only 10% lower than that of normal-weight concrete.
- As assumed, after 28 days of curing, both concretes showed a similar mean compressive strength, determined on cube specimens, 46.5 MPa and 50.7 MPa for LWAC and NWAC, respectively.
- The obtained compressive strength of tested LWAC confirms that achievement of higher strength of lightweight concrete with Certyd aggregate is possible.
- The shape of the stress–strain relationship in the uniaxial compression for the tested lightweight concrete corresponds to the graphs in the tests discussed in the literature. On the other hand, the tested normal-weight concrete, due to the use of dolomite crushed aggregate, showed a slightly different nature of this relationship compared to typical structural NWAC. In the case of normal-weight concrete, up to the level of effort of about 65%, the strain-stress curve was linear, while for lightweight concrete, the corresponding level of effort was 80%.
- As in the case of the compressive strength, the tensile strength value of lightweight concrete at 28 days of curing was 11% lower than that of normal-weight concrete. However, for both concretes, the tensile strength values were slightly lower than those provided in Eurocode EN-1992-1-1 [3]. Nevertheless, these differences are insignificant and prove a good standard estimation.
- The failure of specimens of both concrete types, tested for compressive strength as well as for tensile strength, occurred via the aggregate grains. Such a type of failure indicates that the contact zone in both tested concretes was not the weakest element of their structure and had a positive effect on the homogeneity of these composites.
- As expected, the modulus of elasticity of the tested lightweight concrete was lower (by 34%) than that of normal concrete. It should also be noted that for both LWAC and NWAC, the standard estimations differ only slightly from the obtained test values, which confirms the reliability of the estimations according to Eurocode EN-1992-1-1 [3].
- The increase in compressive strength, tensile strength, and modulus of elasticity between 28 and 90 days of curing is much greater for normal-weight concrete than for lightweight concrete. A smaller increase in the mechanical characteristics of LWAC is caused by the limiting effect of the porous aggregate, which is weaker than the cement matrix.
- The shrinkage of lightweight concrete in the initial period of its curing (up to about 100 days) revealed a much less dynamic development in comparison to normal-weight concrete. The reason for this different rheological behavior of LWAC was probably a significant amount of water accumulated in the porous aggregate. Later, the shrinkage of lightweight concrete increased linearly, but by the end of the tests (i.e., up to 559 days), it did not reach stabilization, and its value was still as much as 36% lower than for normal-weight concrete. Such behavior of tested LWAC turned out to be different from that reported in the available thematic literature, indicating that in the vast majority of cases, the shrinkage of lightweight concrete after about a year reaches the value of shrinkage of NWAC of comparable strength. Due to the lack of shrinkage stabilization for the tested LWAC, it can be expected that it will eventually reach higher values compared to NWAC. However, confirmation of the above would require a much longer observation period.
- The course of shrinkage of the tested normal-weight concrete turned out to be consistent with the estimations of Eurocode EN-1992-1-1 [3], and the determined final values were only slightly higher than those calculated according to the standard. However, the tested final values of shrinkage deformations for lightweight concrete were 38% lower than estimated according to Eurocode EN-1992-1-1 [3], and its course over time differed mainly in the initial period (up to about 100 days).
- The final creep deformations of the tested lightweight concrete were more than two times lower than those of the corresponding normal-weight concrete despite significantly lower final strength.
- The course and values of the lightweight concrete creep coefficient calculated according to EN-1992-1-1 [3] are significantly overestimated in relation to the obtained results of LWAC tests. On the other hand, for normal-weight concrete, the test results practically coincided with the estimated values according to the standard.
- Discrepancies in values of both rheological properties, shrinkage and creep, determined in tests and estimated according to Eurocode EN-1992-1-1 [3] for lightweight concrete may result from the lack or insufficient consideration of the influence of the type of aggregate and the internal curing of LWAC with water accommodated in the aggregate in standard estimations.

The above conclusions from experimental tests allow us to assess that lightweight concrete with Certyd aggregate is characterized by properties, in particular rheological characteristics, suitable for using this material in prestressed structures and can be considered a favorable alternative to normal-weight concrete. In order to verify this thesis, long-term rheological tests were carried out on full-size 6 m long prestressed beams made of both types of concrete. The results of these studies will be the subject of the next publication.

## Figures and Tables

**Table 1:** Properties of lightweight and normal-weight coarse aggregates used for production of concretes.

Property	Certyd 4/16 mm	Dolomite 4/16 mm
Undersize particle content, %	3.7	4.1
Oversize particle content, %	1.5	4.8
Bulk density, kg/m^3^	730	1590
Particle density, kg/m^3^	1380	2640
Water absorption, (WA_24h_), %	19.2	1.4
Maximum water absorption, %	21.8	1.6
Crushing resistance, MPa	7.8	-

**Table 2:** Compositions of concrete mixtures.

Constituent Materials	LWAC	NWAC
	kg/m^3^	kg/m^3^
Aggregate Certyd [4–16 mm]	671	-
Aggregate Dolomite [4–16 mm]	-	1164
Natural sand [0–2 mm]	611	610
Cement CEM I 42.5 R	408	370
Mixing water	145	167
Water for wetting Certyd aggregate	128	-
Superplasticizer SicaViscoCrete 6RS	5.7	5.2

**Table 3:** List of carried out tests used standard procedures and specimens for each concrete type.

Tests	European Standard Procedure	Specimen Type	Number of Specimens
Density	EN 12390-7 [33]	cylinder ϕ150 × 300 mm	12
Compressive strength	EN 12390-3 [34]	cube 150 × 150 mm	18
Compressive strength	EN 12390-3 [34]	cylinder ϕ150 × 300 mm	18
Modulus of elasticity	Method B, EN 12390-13 [35]	cylinder ϕ150 × 300 mm	
Tensile splitting strength	EN 12390-6 [36]	cylinder ϕ150 × 300 mm	18
Shrinkage	-	beam 200 × 200 × 1000 mm	6
Creep			

**Table 4:** Densities of hardened concretes.

Mean Density	LWAC [kg/m^3^]	NWAC [kg/m^3^]
In saturated condition	1980	2320
In natural condition	1890	2270
In oven-dry condition	1740	2170

## Data Availability

Not applicable.
